# Enzymatic activity of variously preserved extracts from spent mushroom substrate (*Pleurotus ostreatus*) and in vitro rumen degradation of extract-treated wheat straw

**DOI:** 10.1186/s40643-026-01071-y

**Published:** 2026-05-29

**Authors:** E. Daniso, P. Comuzzo, M. Spanghero

**Affiliations:** Department of Agricultural, Food, Environment and Animal Science, University of Udine, Via Sondrio 2/a, Udine, UD Italy

**Keywords:** *Pleurotus ostreatus*, Spent mushroom substrate, Ligninolytic enzymes, Neutral detergent fiber, In vitro rumen degradation

## Abstract

White rot fungi obtain nutrients from fibrous substrates through the radical secretion of extracellular ligninolytic enzymes (LE) and spent mushroom substrate (SMS), a by-product of commercial cultivation, is a valuable bioresource of LE. This study aims to compare various techniques for dehydrating and preserving the *Pleurotus ostreatus* SMS extract in terms of enzymatic activity and ability to improve the fiber rumen degradability (NDFD) of extract-treated wheat straw. To convert the extract into stable enzyme powder, it was concentrated before being dried using freeze, spray and vacuum drying processes. The different extracts reconstituted from powders were evaluated for residual LE activity after multiple successive freeze-thaw cycles, as well as their ability to improve wheat straw NDFD. To produce extracts, fresh SMS was suspended in acetate buffer, homogenized and filtered. A portion of the original extract was stored (− 20 °C) and the remainder was concentrated up to 50% of volume by cross-flow filtration. The concentrated extract was divided into four aliquots to be used without processing or spray, vacuum or freeze drying. Extracts were evaluated for total, Mn and lignin peroxidases and laccases (LAs) enzymatic activity and were subjected to four freezing-thawing cycles. Wheat straw was incubated in reconstituted extracts before being tested for in vitro NDFD. Compared to the original extract, reductions of LE were 2–30% in concentrated and 10–36, 4–48 and 74–84% in freeze, vacuum and spray dried, respectively. LE of extracts decreased with the number of freeze-thaw cycles, apart from the spray dried extract. The NDFD of all extract-treated straws resulted higher (*P* < 0.05) than the control, apart from the spray-dried extract and had a high linear relationship with LAs of extracts (R^2^ 0.87, *P* < 0.01). Results support the potential of extract preparations from fungal SMS to be a promising bioresource useful for improving the NDFD of high-fiber forages, such as straw.

**Graphical abstract:**

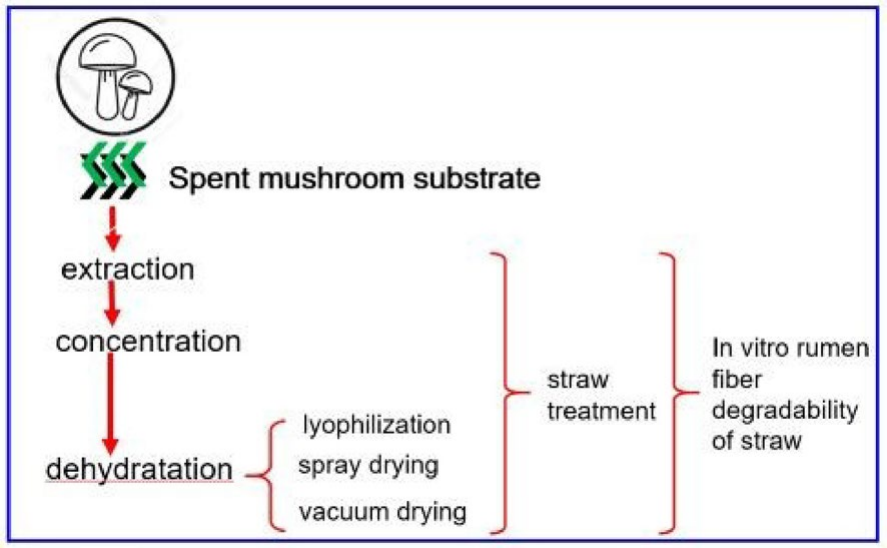

## Introduction

Animals lack digestive enzymes capable of degrading plant fiber components (cellulose, hemicellulose, pectine and lignin), but ruminants can partly utilize fiber through microbial fermentation in the rumen. Lignin, a naturally abundant phenolic polymer in vegetable tissues, is combined with plant fiber to form the complex wall of plant cells and is considered a barrier to a complete utilization of fiber by ruminants because it is resistant to anaerobic microbial attack (Van Soest 1994). The improvement of fiber degradation of forages is an important challenge in ruminant nutrition and among the different strategies the utilization of treatments based on fungal-derived fibrolytic enzymes has received significant attention (Adesogan et al. 2019; Zheng et al. 2025).

White rot fungi are chemo-organotroph organisms which derive their energy from the extracellular break down of polymeric compounds by secreting fibrolitic enzymes via the radical apparatus and absorb monomers to be used as carbon and energy sources (Walker and White 2017). The fungi species of the genus *Pleurotus* belongs to the *Basidiomycota* phylum and are among the most important commercially cultivated mushrooms worldwide. Several enzymes are released in the substrate of cultivation via mycelium (*endoglucanases*,* carbohydrate esterase*,* xilanases*,* cellobiohydrolases*, etc.) and these fungi are also high producers of ligninolytic enzymes (Villani et al. 2025). The main groups of ligninolytic enzymes released in the substrate are manganese peroxidases (MnPs), lignin peroxidases (LiPs), and laccases (LAs), which work together to break down lignin and cellulose (Pérez-Chávez et al. 2022). MnPs, the most prevalent lignin-modifying peroxidases produced by wood-colonizing basidiomycetes and soil-colonizing litter-decomposing fungi, is a glycosylated heme protein secreted in multiple forms, typically with molecular weights between 40 and 50 kDa (Hofrichter 2002). Similarly, LiPs, which are primarily synthesized by white-rot basidiomycetes, are a key extracellular heme peroxidase that play a crucial role in lignin’s oxidative degradation. Characterized as a glycoprotein with molecular weights ranging from 38 to 42 kDa, LiPs exhibit broad substrate specificity and high redox potential, crucial for the initial stages of lignin breakdown (Wariishi et al. 1990).

LAs, multicopper oxidases distributed across fungi, plants, and bacteria, catalyze one-electron oxidations of phenolic compounds and aromatic amines, utilizing molecular oxygen as the electron acceptor. Characterized as glycoproteins with molecular weights between 60 and 90 kDa, LAs employ a mediator system to extend their substrate range to non-phenolic lignin structures. The versatility of laccases in substrate specificity and environmental adaptability makes them key enzymes in lignin biodegradation (Bhardwaj et al. 2022).

While MnPs and LiPs were historically considered the primary lignin-depolymerizing enzymes, LAs are now recognized to contribute collectively to the oxidative breakdown of complex lignocellulosic structures, offering a unifying perspective on lignin biodegradation (Bermek et al. 1998; Higuchi 2004).

Spent mushroom substrate (SMS) from genus *Pleurotus* cultivation is a mix of different plant materials such as straw, cereal grains, twigs and hays on which commercial mushroom are cultivated and represents a substantial and underutilized bioresource of enzymes (Leong et al. 2022) and other bioactive compounds (Akter et al. 2025).

In a prior study, we demonstrated the presence of ligninolytic enzymes in an extract obtained from *Pleurotus ostreatus* (PO) SMS and its efficacy in improving the in vitro rumen degradability of wheat straw fiber (Daniso and Spanghero 2026). However, while existing literature focuses almost exclusively on the immediate efficacy of fresh extracts or SMS, the transition from laboratory-scale discovery to practical farm applications remains hindered by the lack of data on raw extract enzyme stability.

To the best of our knowledge, the current study represents the first systematic effort to address this gap by evaluating different dehydration and preservation methods for SMS-derived ligninolytic enzymes specifically tailored for ruminant nutrition. The primary aim was to compare various techniques, lyophilization (freeze-drying), spray-drying, and vacuum (oven) drying, for converting the raw extract into a stable enzyme powder.

To ensure these bio-additives can be stored and transported under farm conditions without losing their bio-catalytic potential, the reconstituted extracts were evaluated for residual enzymatic activity after multiple successive freeze-thaw cycles. Furthermore, their functional capacity to digest wheat straw fiber was validated through in vitro rumen fermentation, providing a necessary bridge between enzymatic characterization and the development of stable, ready-to-use feed additives.

## Materials and methods

### Extract preparation

The SMS of PO was provided by Consorzio Funghi and Fungamico s.r.l. (Treviso, Italy). To produce the liquid extract, 2 kg of fresh SMS (approx. 30% dry matter (DM)) was suspended in 0.2 M acetate buffer (pH 5.4) in a ratio of 1:2 w/v and homogenized in a C-Tronic 15 Plus apparatus (Sirman, Italy). After 3 h, the mixture was pressed using a pneumatic press (Model A40, Grifo Marchetti, Piadena Drizzona, CR, Italy) to separate the solid from the liquid part and the latter was filtered with a 100 μm sieve to remove large particles in suspension.

About 4 L of clear extract was obtained and a portion (500 mL) was stored at − 20 °C, while the remaining was concentrated up to the 50% of the original volume, using a Sartocon^®^ Slice cross-flow filtration system, fed by a SartoJet four piston pump and equipped with two Sartocon^®^ polyethersulfone ultrafiltration cartridges (Sartorius Stedim, Antella, FI, Italy; MWCO: 10 kDa; total filtration area 0.2 m^2^).

### Dehydration of the concentrated SMS extract

To investigate the effect of the drying process on enzyme activity, the concentrated liquid was divided into four portions of about 500 mL each one, which were further processed as follows: (i) one aliquot has been used as it was, without processing; (ii) another was subjected to freeze drying; (iii) the third was treated by spray drying and (iv) the last was dried under vacuum.

Freeze drying was carried out using a Christ Epsilon 2–4 LSC-plus lyophilization system (Del Tek, Italy) with the following setup: a rapid freezing step for 20 min at a set temperature of − 30 °C, then a thermal ramp increasing the temperature in 10 °C increments, until a final temperature of 20 °C was reached. Thermal ramp was as follows: − 20 °C after 8 h, − 10 °C after 16 h, 0 °C after 8 h, 10 °C after 16 h, and finally 20 °C after 8 h. The set conditions were monitored and displayed via a touchscreen interface, which provided real-time tracking of all process parameters. The heating phase was conducted under a vacuum pressure of 0.2 mbar, achieved using a vacuum pump. This condition facilitated the removal of water in vapor form through sublimation, which was simultaneously trapped as ice by refrigerated coils.

Spray drying process took about three hours; the concentrated extract was processed at 140 °C and a vacuum range of 35–40 mmHg; the sample feed rate was approximately 3.5 mL/min.

Concerning vacuum drying, the concentrated extract was put in aluminum baking cups and introduced in a vacuum drying oven. Initial conditions were as follows: temperature 62 °C and pressure 1 bar for 24 h. However, due to the high adhesion of the samples to the cups, the protocol was changed and the time reduced to 18 h; in this way, the extract was not dried completely but the water was reduced by 90%.

The unprocessed concentrated liquid extract and the powders obtained from the different drying methods were stored at − 20 °C until their use for the experimental activities described below.

### Enzymatic assay on extracts (experiment 1)

The different extracts were reconstituted from powders in distilled water at a uniform initial concentration and were evaluated in triplicate for the different residual activity of total peroxidases (TPs), MnPs, LiPs and LAs.

The Peroxidase Assay Kit (Sigma-Aldrich, Germany) was used for the determination of the TPs, following the manufacturer’s instructions. The absorbance was measured at 570 nm using the microplate reader SUNRISE (Tecan, Germany) and for the calculation of the peroxidase activity was used the following formula:

$$\begin{aligned}&Peroxidase \;activity\; (U/L)\;\\&= \;(RSample - RBlanck)/(RResorufin - RH2O) \\&\times [Resorufin](\mu M) \times n\end{aligned}$$

 

where: R*Sample*, R*Blank*, R*Resorufin* and R*H2O* are optical density readings of the Sample, Sample Blank, Resorufin and Water respectively, n is the dilution factor and the [Resorufin] is 50 µM for colorimetric assays and U/L the enzyme units.

MnPs activity was assessed by oxidation of 10 µL at 0.5 mM of 2,6-dimethoxyphenol in 160 µL of 0.1 M malonate buffer (pH 4.5), with 10 µL of 1mM MnSO_4_, 5 µL 0.5 mM of H_2_O_2_ and 10 µL sample extract in an ELISA microplate well (Ten Have et al. 1998).

LiPs activity was assessed by oxidation of 10 µL at 2 mM of 3,4-dimethoxyphenyl alcohol to veratraldehyde, in 180 µL of 0.1 M tartrate buffer (pH 3), with 5 µL 0.4 mM H_2_O_2_ and 10 µL sample extract in an ELISA microplate well (Camarero et al. 1999).

LAs activity was determined using 2,2’-azino-bis(3-ethylbenzothiazoline-6-sulfonic acid, ABTS) as a substrate. A 100 µL aliquot of the supernatant from each experimental sample was mixed with 1 mL of 0.1 M acetate buffer (pH 4.5) and 250 µL of ABTS. Subsequently, 200 µL of this reaction mixture was transferred to an ELISA microplate and incubated at 25 °C for 15 min, as described by Ghose et al. (2023).

The absorbance of the different reaction mixtures was measured using the microplate Infinite^®^ 200 PRO reader (Tecan, Italy), and enzyme activity, expressed in enzyme units (U/L), was calculated using the following formula:

$$\begin{aligned}&Enzyme\; activity\; (U/L) \\&= (\Delta OD \times V1)/(\varepsilon \times V2 \times \Delta t) \times 10^6\end{aligned}$$

where: ΔOD is the difference in absorbance between the initial and final readings, V1 is the total volume of the enzyme reaction mixture, V2 is the volume of the enzyme solution, ε is the molar absorption coefficient of the reactive reagent (2,6-dimethoxyphenol, 3,4-dimethoxyphenyl alcohol and ABTS) and Δt is the reaction time.

### Enzymatic shelf-life of extracts after sequential freeze-thaw cycles (experiment 2)

Extract samples (original extract, concentrated original extract, and concentrated original extracts dried using the three drying techniques) were subjected to four consecutive freezing-thawing cycles (each lasting one week) before being prepared at a uniform initial concentration for comparison purposes. To assess the impact of freezing-thawing cycles on enzymatic activity, LAs was chosen as the representative enzyme for this examination because it demonstrated adequate consistency of activity across multiple treatments in Experiment 1 (with the exception of the spray dry treatment). LAs activity was then quantified after each cycle and initial samples were also stored for two months and subsequently analyzed for residual LA activity, before being utilized for the wheat straw treatment, to evaluate the enzyme shelf-life.

### Rumen in vitro NDF degradability of treated-extract wheat straw (experiment 3)

To evaluate the ligninolytic activity the treated extracts were added with distilled water to reach the expected enzymatic concentration of the original extract (uniform initial concentration). Subsequently, 4 g of milled straw (88.6% DM; neutral detergent fiber (NDF) content: 76.7% DM) was placed in a beaker with a magnetic stirring bar and submerged in 200 mL of the respective five test extracts (original extract, concentrated original extract, and concentrated original extracts dried using the three drying techniques) and in distilled water used as control. To activate the heme-presenting enzyme class, such as MnPs and Lips, 10 µL of H_2_O_2_ was added to each beaker which were then incubated at 25 °C for 48 h on a multi-position magnetic stirrer (IKA RO10, IKA, Germany). After incubation, the straw was separated from the corresponding extract by centrifugation and the residual extract was removed after two sequential washing steps with Milli-Q water, using the same centrifugation method. The residual straw samples were dried overnight at 60 °C and then re-equilibrated to room conditions for 24 h.

**Fig. 1:**
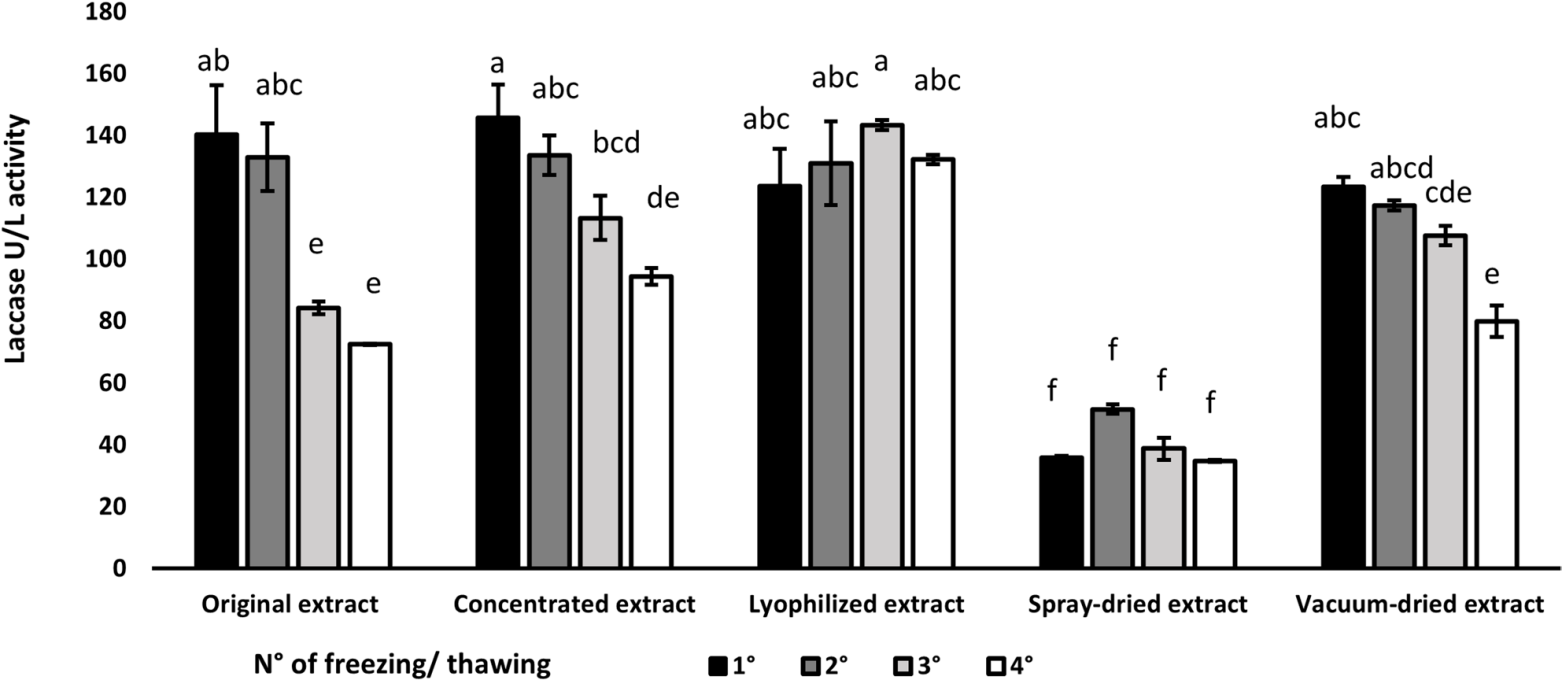
Laccase activity of extracts from Pleurotus ostreatus spent mushroom substrate obtained by simple extraction (original) or after concentration (concentrated) and different drying methods (lyophilization, spray drying and vacuum drying) and after four freeze-thaw cycles (bars represent means ± standard deviation and different letters indicate significant differences, *P* < 0.05; residual means square error: 7.05)

In vitro neutral detergent fiber degradability (NDFD) was determined by incubating Ankom F57 filter bags containing 250 mg of ground wheat straw in diluted rumen fluid using the Ankom Daisy II Incubator (Ankom Technology Corporation, New York). Incubations lasted 48 h at 39 °C in jars containing 400 mL of filtered liquid rumen and two buffer solutions (266 mL of solution A: KH_2_PO_4_ 10 g L^−1^, MgSO_4_ 7H_2_O 0.5 g L^−1^, NaCl 0.5 g L^−1^, CaCl_2_ 2H_2_O 0.1 g L^−1^, Urea 0.5 g L^−1^; 1330 mL of solution B: Na_2_CO_3_ 15.0 g L^−1^, Na_2_S 9H_2_O 1.0 g L^−1^, Spanghero et al. 2010). Rumen fluid was collected from healthy culled dairy cows immediately after commercial slaughter and brought to the laboratory in sealed glass vials that were flushed with CO_2_ and kept at 39 °C for 30 min. Following the incubation, the bags were removed from the jars, cleaned with deionized water, and dried overnight at 105 °C. The NDF content of the bags was analyzed using the AnkomII Fiber Analyzer (Ankom Technology Corporation, New York), in accordance with the method described by Mertens (2002). Three filter bags were used for each straw treatment (original extract, concentrated original extract, and concentrated original extracts dried using the three drying techniques) and the test was replicated in two independent rumen fermentation runs, which used rumen liquids collected at the slaughterhouse from different animals (Fig. 1).

### Statistical analysis

Statistical analysis used the procedures of the Statistical Analysis System (SAS Institute, 2019). In all the experiments the effect of different extract was included as fixed effect (α, i = 1,5) and Y, µ and ε are the experimental trait analyzed, the overall mean and the random residual error, respectively. The normality of variance was tested using the tests of Proc Univariate of SAS (Kolmogorov-Smirnov, Cramer-Von Mises and Anderson-Darling). The Tukey Multiple Comparison Test was used to determine significant differences between the means of multiple groups.

In Experiment 1, TPs, MnPs, LiPs and LAs enzyme activities were analyzed according to the following factorial linear model:

$$Yij=\mu + \alpha i + \varepsilon ij$$

In Experiment 2, LAs activity of different extracts after progressive freezing-thawing cycles were analyzed with the following linear model:

$$Yijk = \mu + \alpha i + \beta j + (\alpha \beta)ij + \varepsilon ijk$$

where β is the effect of number of freezing-thawing cycles (j = 1,4) and αβ is the interaction between the effect of the extract and the number of freezing-thawing cycles.

In Experiment 3, the NDFD of straw previously incubated in different extracts were analyzed with the following linear model:

$$Y_{ijk} = \mu + \alpha _{i} + \beta _{j} + \varepsilon _{ijk}$$

where β_j_ is the random effect of fermentation run (block,  j = 1,2).

**Fig. 2:**
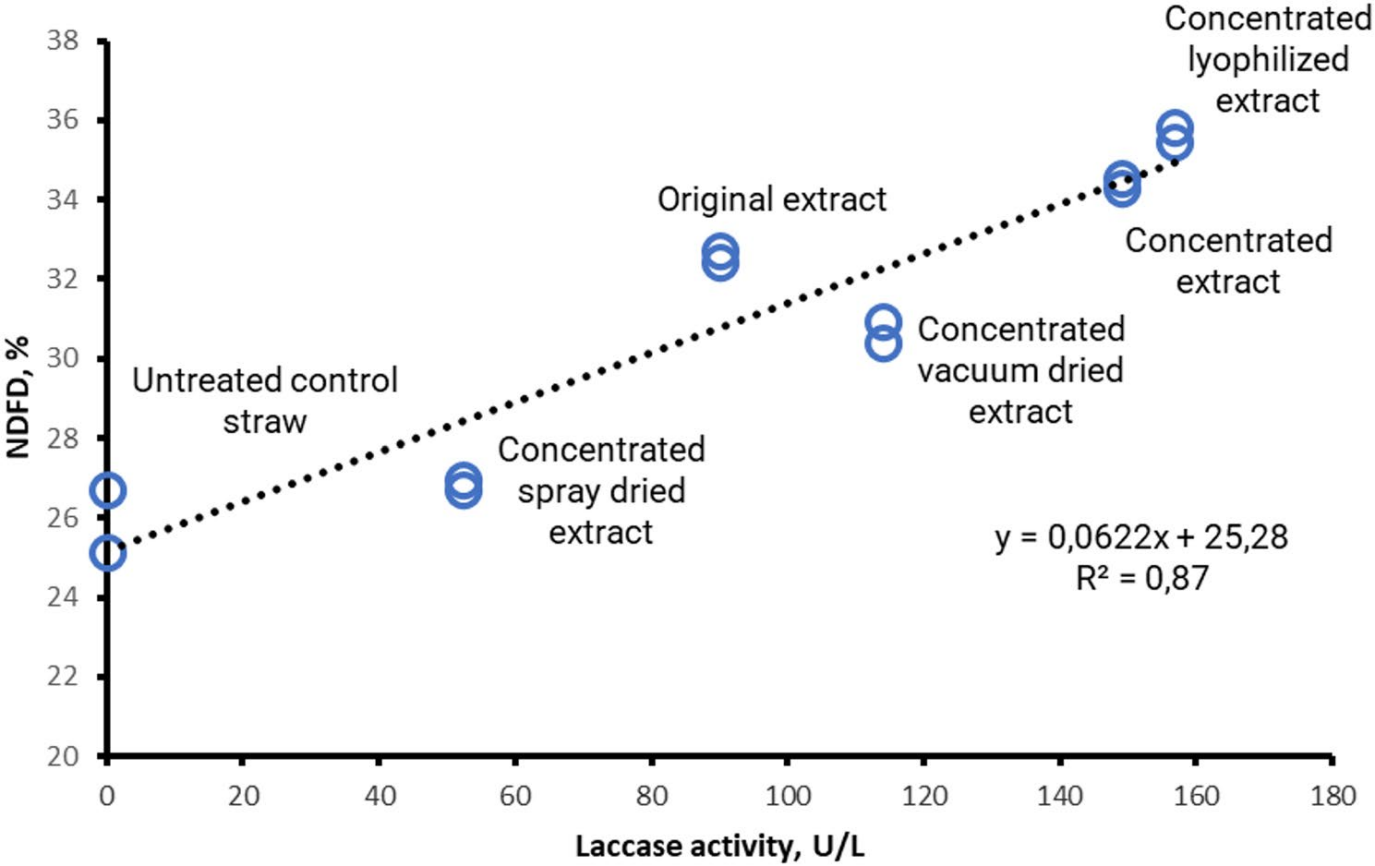
Relationship between the NDFD of wheat straw and the LAs activities of extracts used to treat the straw (*P* < 0.01)

In Experiment 3 the NDFD of the extracted treated straws was also regressed on LAs activity of the extracts according to the following linear mixed model of SAS:

$$Yij = \beta 0 + \beta 1Xij + si + \varepsilon ij$$

where:

Yij is the NDFD of straw observed for the LAs activity j (j = 1,6) in the fermentation run i (i = 1,2); β0 is the overall intercept across fermentation runs (fixed effect), β1 is the overall regression coefficient for the linear effect of x (fixed effect), Xij is the LAs activity j in fermentation run i, si is the random effect of fermentation run i, approximately normal (0, σ2s), and εij is the residual error, approximately normal. Homoscedasticity was verified by the White’s test and adjusted values for the fermentation rumen fluid, calculated according to St-Pierre (2001), were used to generate the two-dimensional graph in Fig. 2.

## Results

### Experiment 1: Enzymatic activity of extracts

Table 1 shows the enzymatic activity of ligninolytic enzymes in different extracts. The TPs activity.

**Table 1:** Enzymatic activity of extracts from *Pleurotus ostreatus* spent mushroom substrate obtained by simple extraction (fresh) or after concentration (concentrated) and further drying by different methods (lyophilization, spray drying and vacuum drying)

	Fresh^1^	Concentrated^2^			
		Fresh	Drying methods^3^		
			Lyophilized	Spray dried	Vacuum dried
Total^4^ P, U/L	609.6^a^	598.9^a^	551.3^ab^	86.9^c^	506.1^b^
Manganese^4^ P, U/L	63.3^a^	53.1^a^	39.9^b^	10.9^c^	34.3 ^b^
Lignin^4^ P, U/L	60.3^a^	42.7^b^	38.8^bc^	9.4^d^	28.8 ^c^
Laccases^4^, U/L	140.2^a^	145.8^a^	120.7^a^	35.3^b^	134.4 ^a^

^1^Measured after 1 d of freezing;

^2^Reduction of 50% of volume with respect to the original fresh extract

^3^From 500mL of concentrate extract to 19.6 g, 18.9 g and 50 mL after the three drying methods (lyophilization, spray drying and vacuum drying, respectively)

^4^Means result from 3 analytical replicates. Means in the same row with different superscripts (a, b, c, d) are statistically (*P* < 0.05) different (Residual means square errors of 23.32, 4.15, 4.07 and 11.35 for total peroxidase, MnP, LiP and Las, respectively)

was not significantly different between the original and the concentrated original extract, both showing the highest activity (609.6 and 598.9, U/L). The activity slightly decreased for the lyophilized extract (− 8%) and was much lower for the vacuum dried (− 15%, *P* < 0.05) with the lowest value for the spray-dried (86.9 U/L, about 15% of the original extract, *P* < 0.05).

The original extract had the highest MnPs activity (63.3 U/L), with the concentrated original extract showing a 20% reduction: the lyophilized and vacuum dried extracts showed reductions (*P* < 0.05) of about 50% compared to original extract and the lowest residual activity was for the spray-dried extract (10.9 U/L, about 17% of the original extract). Regarding LiPs activity, the original extract showed the highest activity (60.3 U/L), followed by the concentrated original extract (42.7 U/L, *P* < 0.05). The lyophilized extract had LiP contents intermediate between the concentrated and the vacuum-dried extract (28.8 U/L), while the spray-dried extract displayed the significantly lowest LiP activity (about 16% of the original extract, *P* < 0.05). The LAs activity varied between 120.7 and 145.8 U/L without significant differences among extracts with the exclusion of the spray-dried extract which showed a significantly lower activity (35.3 U/L, *P* < 0.05).

### Experiment 2: Freezing-thawing of extracts and enzymatic activity

Extracts obtained from Experiment 1 were subjected to four consecutive freeze-thaw cycles, with LAs activity measured after each cycle. The interaction between type of extract and the number of freeze-thaw cycles was significant (*P* < 0.01) and results are shown in Fig. 1.

Spray dried extracts showed no modification among freeze-thaw cycles and had lower concentrations (*P* < 0.05) compared to other treatment combinations. LAs activity in the original, in the concentrated original and in the vacuum dried extracts decreased consistently as the number of freeze-thaw cycles increased. However, there were differences in the decreasing steps: the reductions from the first to the second cycle were never statistically significant, whereas the reductions from the second to the third cycle were significant for the concentrated extract. For all extracts, there were no statistical differences between the third and the last freeze-thaw cycle.

Lyophilized extracts did not decrease as the number of freeze-thaw cycles increased, and the variations between freeze-thaw cycles were not statistically significant.

### Experiment 3: In vitro rumen fermentation

Table 2 shows the in vitro rumen fermentation results of wheat straw treated with various extracts

**Table 2:** Laccases enzymatic activity of extracts from *Pleurotus ostreatus* spent mushroom substrate obtained by simple extraction (fresh) or after concentration (concentrated) and further drying by different methods (lyophilization, spray drying and vacuum drying) and in vitro rumen NDF degradability of wheat straw treated with extracts

	Control	Fresh^1^	Concentrated^2^			
			Fresh	Drying methods^3^		
				Lyophilized	Spray dried	Vacuum dried
NDFD^4^, %	25.9^d^	32.6^ab^	34.4^a^	35.6^a^	26.8^d^	30.7^b^
Laccases^4^, U/L	–	90.1^c^	148.9^a^	156.9^a^	52.3^d^	113.9^b^

^1^Measured after 1 d of freezing;

^2^Reduction of 50% of volume with respect to the original fresh extract

^3^From 500mL of concentrate extract to 19.6 g, 18.9 g and 50 mL after the three drying methods (lyophilization, spray drying and vacuum drying, respectively)

^4^Means of NDFD result from 6 analytical replicates (3 replicates within each of 2 fermentation runs. Laccase means result from 3 analytical replicates. Means in the same row with different superscripts (a, b, c, d) are statistically different (Residual means square errors of 1.76 and 7.87 for NDFD and LAs, respectively)

treatments, including the untreated straw (control), and the LAs activity of the extracts. The in vitro rumen fermentation was performed with extracts stored at − 20° C for 60 d and the LAs were measured to monitor the actual activity. There was a reduction in comparison with the one measured 1 day after the first freezing due to prolonging the preservation period freezing for the original (from 140.2 to 90.1 U/L, *P* < 0.05) and vacuum dried (from 134.4 to 113.9 U/L, *P* < 0.05), but not for the concentrated extract (statistics not in table). Both for lyophilized and spray dried extracts there were apparent increases of the activity. In general, the LAs activities assessed at one and sixty days of freezing (Tables 1 and 2, respectively) revealed a good consistency in ranking between treatments. In contrast, MnP and LiP were excluded from the regression analysis due to their intrinsic instability during freeze-thaw cycles. In fact, the resulting enzymatic activities after sixty days were negligible or below the detection limit, thus preventing any significant statistical correlation or comparative evaluation.

Wheat straw treated with the concentrated fresh and the concentrated lyophilized extract had the highest NDFD followed by that treated with the fresh extract. The straw treated with the vacuum-dried extract had a more degradable fiber than the spray-dried straw, which had a NDFD comparable to that of the untreated straw.

The relationship between the NDFD of wheat straw and the LAs activities of extracts showed a very good relationship, as shown by Fig. 2 (R^2^ 0.87, *P* < 0.01).

## Discussion

In a previous study, we demonstrated that the SMS extract form *Pleurotus ostreatus* was effective in improving rumen degradation of wheat straw fiber, while the present study compares various techniques for dehydrating and preserving the *Pleurotus ostreatus* SMS extract in terms of LE activity and ability to improve the fiber rumen NDFD of extract-treated wheat straw. The aim is to get further gross information to orient a subsequent evaluation of scaling up the process at an industrial level.

### Technological extract preparation and enzyme activity

The concentration process is a pretreatment to improve efficiency of the subsequent drying process. In our conditions, the process allowed the elimination of about 50% of water and the enzymatic activity demonstrated a relatively mild effect on total peroxidases and laccases, while it determined a significant loss for manganese and lignin peroxidases (− 16 and − 30%, respectively).

Freeze-drying and vacuum drying were able to further remove 96 and 90%, respectively, of the water of the concentrated liquid and both processes determined around 15–20% loss of enzymatic activity for total peroxidases and laccases and around 30–40% for manganese and lignin peroxidases.

In contrast, spray drying, which removed approximately 96% of the water from the concentrated liquid, resulted in a significant drop in activity of all enzymes (from 75 to 90%).

This observed reduction in enzyme activity following spray drying is likely attributable to the specific technological demands of the process. The nebulization of the extract at a temperature of 140 °C, may have denatured the enzymes, due to the rapid moisture loss and the exposure to thermal stress during the drying process. The other treatments, which do not involve high pressure or elevated temperatures, exhibited a less pronounced effect on enzyme activity.

However, to overcome the protein denaturation problem, different bio stabilizers (e.g., sugars, amino acids) could be used as encapsulating agents to preserve enzymes activity and functionality during the spray drying process; nevertheless, the current study focused solely on a base process without additions of preservatives or protecting agents (Ameri and Maa 2006).

Notably, laccase activity was not significantly different between the original extract and the concentrated, freeze-dried and vacuum dried extracts. Consequently, it was selected as a reference enzyme for further analysis regarding freeze-thaw cycles.

### Freeze-thaw effect on enzyme activity

The impact of freeze-thaw cycles on enzyme activity varied significantly across different preservation methods. Specifically, both lyophilized and spray-dried extracts exhibited minimal changes in enzymatic activity derived from the freeze-thaw cycles, indicating effective preservation. However, the consistently lower activity observed in the spray-dried extract suggests potential partial enzyme degradation due to the specific technological treatments applied, as previously underlined. In contrast, the untreated original extract showed a marked decrease in activity, suggesting its vulnerability to freeze-thaw stress, probably linked to its greater water content. The concentrated extract displayed a reduced sensitivity to these cycles in comparison with the original extract, potentially due to an increased protein concentration resulting from partial water removal. Similarly, the vacuum-dried extract demonstrated a reduction in enzymatic activity following a similar kinetic pattern to the other two extracts, which could be attributed to residual moisture. This incomplete drying in the vacuum-dried procedure, which contributed to enzyme instability, was needed because complete desiccation resulted in sample adhesion to the holder, complicating its recovery and making the process impractical. These results highlight the importance of moisture control in enzyme preservation, a finding consistent with Dianawati and Shah (2011), who emphasized the critical role of moisture content in enzyme stability. Therefore, achieving complete drying, as demonstrated by lyophilization and spray drying methods, is crucial for preserving enzyme activity during freeze-thaw cycles. Moreover, while spray drying requires a difficult parameter optimization with the need of a bio stabilizer (Wang and Selomulya 2020), lyophilization seemed a robust and easy method for enzyme preservation under fluctuating temperature conditions, as corroborated by Prado et al. (2022).

### Rumen in vitro NDF degradability of wheat straw treated with extracts 

Wheat straw is frequently utilized in scientific literature to evaluate the efficacy of fiber degradation by fungi (direct inoculation or extract treatment), and in vitro rumen degradation assays are commonly employed to assess improvements in fiber digestibility (Martens et al., 2023; Rodrigues et al., 2008). The straw NDFD was lower than tabulated value (NRC, 2021; Dairy One laboratory, Ithaca, NY, 2026) and this could be the result of mode of calculation (e.g., residual ash included or not) and by the method of determination (direct in vitro determination or estimated by NIR). A further reason could be the fact that straw inserted in filter bags was treated (in extracts or in water) before to be incubated and this might have packed the material and reduced the accessibility of rumen bacteria. However, these conditions could have affected the whole accuracy but not the precision in the relative comparisons between treatments.

In our study, wheat straw incubated with *Pleurotus ostreatus* SMS extracts concentrated and lyophilized showed noticeably higher NDFD, with increases of around 35% relative to the untreated straw control (Table 2; Fig. 2). Furthermore, all other treatments demonstrated an increase in NDFD depending on their LAs enzyme content, reinforcing the functional role of this enzyme, consistent with our previous findings (Daniso and Spanghero 2026). However, the extracts comprise a combination of various ligninolytic enzymes, as demonstrated in Experiment 1, and the positive effect on fiber degradation is the result of several enzymatic actions. The satisfactory relationship between NDFD and LAs supports the use of this enzyme as indicator of the ligninolytic activity of fungal extracts.

Despite the extracts being prepared through precise dilutions to ensure a uniform expected enzyme content, thereby mimicking the original (untreated) extract for rigorous comparative analysis, variations in absolute laccase activity, utilized as a reference enzyme, were observed across the different extracts (Fig. 2). These discrepancies can be attributed to the specific technological treatments applied during extract processing, coupled with potential degradation during two months of prolonged storage at − 20 °C. Notably, the original extract showed a 35% reduction in laccase activity, decreasing from 140.2 to 90.1 U/L. This significant decrease is likely a direct consequence of its inherent high-water content and comparatively lower protein concentration, factors that make it more susceptible to cryoconcentration effects. Cryoconcentration, a phenomenon occurring during freezing, involves the preferential crystallization of water, leading to an increased concentration of solutes in the remaining liquid phase. In a concentrated solution, this process results in a relatively moderate increase in solute concentration compared to the initial state. Conversely, in a dilute solution, such as the original extract, cryoconcentration can result in a dramatic increase in solute concentration (Cao et al. 2003). This abrupt augmentation creates an extreme chemical environment, characterized by elevated ionic strength, altered pH, and increased solute-solute interactions, which can induce denaturation or other forms of damage to enzymes present in the original extract. In contrast, the other extracts, subjected to technological treatments that reduced moisture content and increased protein concentration, did not exhibit such drastic reductions in laccase activity following prolonged storage. This observation is in line with the idea that lower moisture and higher protein concentrations mitigate the adverse effects of cryoconcentration, thereby preserving enzyme stability, as previously reported (Dianawati and Shah 2011).

### Limitations of the study

The findings of this study provide a solid foundation for enzyme preservation strategies, yet several limitations must be acknowledged to contextualize the results. Concerning the comparison of different technologies, the data indicate a significant loss of enzyme activity due to thermal stress during the spray-drying process. This highlights the need to select appropriate encapsulating agents to preserve structural integrity, as well as to standardize the optimal ratio between encapsulating material and extract powder.

Furthermore, it should be noted that this study focused exclusively on enzymatic activity and fiber degradability, without performing a detailed protein quantification or an enzyme purity assessment. While the crude extract effectively represents a realistic “farm-ready” product, future biochemical characterizations could clarify the specific contribution of individual isoforms to the observed results. Similarly, while we assessed the ligninolytic action of the extracts on wheat straw, the current experimental design did not include a microbial analysis of the straw after treatment; such an evaluation would be essential to rule out any competitive or synergistic effects with the native straw microbiota. From an application perspective, this study has partial practical on-farm relevance as it was conducted at a laboratory scale using controlled procedures. The absence of in vivo validation remains a primary limitation; while in vitro rumen fermentation is a robust proxy, animal trials are required to confirm the actual impact on intake, digestibility, and zootechnical performance. Additionally, the temporal scope of this research was limited to a 60-day freezing period; therefore, the long-term stability and shelf-life of these dehydrated powders beyond this window remain to be established.

Finally, to bridge the gap between laboratory success and industrial adoption, this study must be integrated with a comprehensive economic feasibility examination. A conventional financial analysis, based on a structured cash flow, is required to compare the different technologies (lyophilization, spray-drying, and vacuum-drying) in terms of economic efficiency, labor capacity, and apparatus costs. Addressing these limitations through future multidisciplinary research will be key to transitioning SMS-derived enzymes from a promising biotechnology to a standard tool in ruminant nutrition.

## Conclusions

The present study conclusively demonstrates that a sequential processing strategy, incorporating a filtration step to reduce extract water content followed by lyophilization, effectively yields a stable ligninolytic enzyme powder mix with sustained enzymatic activity from *Pleurotus ostreatus* SMS.

Results not much lower were found for vacuum dried, while the worst residual enzymatic in spray drying extracts needs the application of protective bio stabilizers. This optimized enzymatic product, when applied to wheat straw, resulted in significant enhancement of in vitro fiber degradability, achieving a maximum + 35% increase. In general, when the processes were able to maintain a good residual enzymatic activity, these findings highlight the potential of these extracts for improving the digestibility of high-fiber agricultural by products. Building upon these promising in vitro results, future research endeavors will focus on assessing on the scaling-up, the practical applicability of this enzyme formulation in on-farm settings. Specifically, investigations will be conducted to evaluate its efficacy in enhancing the quality and digestibility of silage derived from high-fiber feedstocks.

## Data Availability

The data that support the findings of this study are available from the corresponding author upon reasonable request.
